# Primary Squamous Cell Carcinoma of the Ovary

**DOI:** 10.7759/cureus.5884

**Published:** 2019-10-10

**Authors:** Zain Abid, Maham Fatima, Desaar Zehra, Masooma Abid, Salauddin A Khan

**Affiliations:** 1 Oncology, Jinnah Postgraduate Medical Center, Karachi, PAK; 2 Internal Medicine, Jinnah Sindh Medical University, Karachi, PAK; 3 Internal Medicine, Army Medical College, Rawalpindi, PAK; 4 Medicine, Jinnah Medical and Dental College, Karachi, PAK; 5 Medicine, United Medical and Dental College, Karachi, PAK

**Keywords:** primary squamous cell carcinoma, mature cystic teratoma, endometrioma, brenner tumor

## Abstract

Primary squamous cell carcinoma (SCC) is a rare entity that usually arises from the malignant transformation of a mature cystic teratoma, an endometrioma, or a Brenner tumor. The de novo occurrence of the pure variety in the absence of a prior lesion is the rarest type, and it arises from the metaplasia of the surface epithelium of the ovary. Because of its rarity, a definitive treatment protocol for treatment is not yet available. We present a case of pure primary SCC of the ovary that was managed by surgery followed by chemotherapy.

## Introduction

Ovarian neoplasms are composed of epithelial, germ cell, and sex cord stromal differentiation [1]. Pure primary ovarian squamous cell carcinoma (SCC) has an extremely rare incidence. SCC is strongly associated with pre-existing ovarian lesions, such as dermoid cysts, Brenner tumors, or endometriosis [2]. Due to the rarity of the pure variety of SCC, effective treatments are yet to be discovered. The stage of the tumor is solely responsible for the prognosis. Patients with early stage pure primary ovarian SCC may remain disease-free subsequent to tumor debulking. However, those patients with advanced-stage disease may experience a poorer outcome despite treatment with postoperative chemotherapy and/or radiotherapy. The present case study describes a patient with pure primary ovarian SCC and presents a review of the literature. Written informed consent was obtained from the patient’s family.

## Case presentation

A 35-year-old female presented to the Department of Clinical Oncology at the Jinnah Postgraduate Medical Centre in Karachi with complaints of mild, persistent pain in the lower abdomen for six months and vaginal discharge sometimes mixed with blood for two months. Her menstrual cycles were normal. A gynecological examination revealed a 4 cm × 5 cm bilobed, firm-hard, tender mass with restricted mobility. The results of her hematological and biochemical tests and her chest X-ray were within normal limits. The CA-125 level was within normal limits, that is 6 IU/L. An ultrasound of the pelvis revealed an echogenic mass in the midline of the pelvis with irregular margins measuring 5.5 cm × 4.1 cm.

Exploratory laparotomy was done and the appendix, uterus, cervix, right tubo-ovarian mass, and left tube and ovary were removed. A histopathology report revealed a right-sided ovarian mass of 10 cm × 8.5 cm × 5 cm, which was moderately differentiated with occasional foci of glandular differentiation and extensive necrosis. No areas of Brenner tumor or endometroid tumor were seen on further sampling of the tissue. The tumor also involved the appendix. The right fallopian tube, left ovary, left fallopian tube, and uterus were unremarkable. Immunohistochemical markers were performed, which showed CK 5/6 positive and p63 positive in tumor cells. Thus the final diagnosis of primary squamous cell carcinoma pT4b (IVB) was made.

A magnetic resonance imaging (MRI) of the pelvis revealed a large soft tissue mass measuring 4.5 cm × 5.8 cm in the pelvis in the midline, which most likely represents a residual/recurrent previously noted tumor mass of the ovary. There was also evidence of a few subcentimeter lymph nodes seen in the iliac regions bilaterally. A computed tomography (CT) scan of the chest showed that there was no distant metastasis to the lungs.

Chemotherapy with carboplatin 600 mg and paclitaxel 175 mg for three weeks was started. Three cycles of chemotherapy resulted in only 50% reduction of the clinically palpable right pelvic mass as assessed by a CT scan. The patient and her family were prognosticated about the poor response and survival.

## Discussion

Primary ovarian SCC is a rare entity, with the majority of cases preceded by dermoid cysts. On the other hand, primary ovarian SCC may also be associated with Brenner tumors and endometriosis [2]. The de novo occurrence of the pure primary form is the rarest, which results from the metaplasia of the surface epithelium of the ovary. The incidence of primary SCC of the ovary is less than 2%, with the majority of the cases developing from the malignant transformation of a mature cystic teratoma (MCT) and also from endometriosis or a Brenner tumor [3]. MCT is one of the most common benign ovarian tumors and has an incidence of malignant transformation of about 1%-2%. The most common transformation in an MCT is SCC, although adenocarcinoma, sarcoma, and carcinoid have also been rarely reported [4]. Ovarian SCCs arising within a dermoid cyst appear incidentally on histology. A primary ovarian SCC originating from the malignant transformation of a dermoid cyst has an incidence of approximately 2%. According to a study, 2.5% of the metastatic ovarian tumors are of squamous cell type, with the majority being directly extended from the cervix. Moreover, the most significant association with the reported cases of pure ovarian SCC is with cervical dysplasia [2]. The prognosis of SCC arising from MCT depends solely on the stage of the disease. According to a report, Kikkawa et al. stated that stage I and stage II patients had a five-year survival rate of 95% and 80%, respectively, whereas stage III and IV patients had a five-year survival rate of 0%. However, many suspicious morphological changes are identified during follow-up, and as a result, over 60% of the cases are identified at stage I or II of SCC. Moreover, such transformations are also occasionally identified during the postoperative pathological examination of the resected MCT tumors that were originally considered to be benign [4].

According to a case report that studied a total of 37 cases of ovarian primary SCC, 19 were associated with a dermoid cyst (SCCD), seven were associated with endometriosis (SCCE), 11 were pure (SCCP), and the last 18 cases fell within the new World Health Organization category of SCC in the surface epithelial-stromal category. The 11 patients with SCCP were 27-73 (mean, 56) years old. The tumors were 6 cm-26 cm in greatest diameter, usually solid with focal necrosis. Three patients with SCCP also had cervical squamous cell carcinoma in situ. The patients with SCCE had a poorer overall survival than those with SCCD. Five of the six patients with SCCE died of their disease despite the adequate follow-up (mean survival, five months); also, in all five cases of SCCE reported in the literature, the patients died of their disease (mean survival, four months). The stage of the tumor and its grade correlated best with overall survival for all three types of SCC [5].

Primary squamous cell carcinoma of the ovary has a poor prognosis [6]. Also, because of its rarity and its similarity to MCT, the preoperative treatment is not yet known. Therefore, a study was done to assess the value of tumor markers and clinical characteristics to distinguish between MCT and SCC arising from MCT. Furthermore, the age and tumor size are important factors in making a differential diagnosis. Therefore, SCC and carcinoembryonic antigen (CEA) levels should be measured in patients aged 45 years or older who have an MCT-like ovarian tumor larger than 99 mm in greatest dimension [7].

The survival rate of patients with advanced stage primary squamous cell carcinoma of the ovary is dismal and effective treatment is unknown [8]. However, a remarkable response of this tumor to weekly paclitaxel-carboplatin has been observed, which is also a safe and effective treatment for advanced tumors with resistance to chemotherapy [6].

## Conclusions

The main purpose of reporting this case is to create awareness about this rare tumor that has an extremely poor prognosis. To summarize, primary SCC of the ovary is a rare tumor with limited data and no definitive treatment protocols. Therefore, research should be done to prepare the appropriate protocols for managing such cases.
